# Features and treatment modality of iliopsoas abscess and its outcome: a 6-year hospital-based study

**DOI:** 10.1186/1471-2334-13-578

**Published:** 2013-12-09

**Authors:** Ming-Shun Hsieh, Shih-Che Huang, El-Wui Loh, Che-An Tsai, Ying-Ying Hung, Yu-Tse Tsan, Jin-An Huang, Lee-Min Wang, Sung-Yuan Hu

**Affiliations:** 1 Department of Emergency Medicine, Taichung Veterans General Hospital, Taichung, Taiwan; 2 School of Medicine, Chung Shan Medical University, Taichung, Taiwan; 3 Institute of Medicine, Chung Shan Medical University, Taichung, Taiwan; 4 Kaohsiung Municipal Kai-Syuan Psychiatric Hospital, Kaohsiung, Taiwan; 5 Division of Infectious Disease, Department of Internal Medicine, Taichung Veterans General Hospital, Taichung, Taiwan; 6 Department of Radiology, Taichung Veterans General Hospital, Taichung, Taiwan; 7 Department of Health Service Administration, China Medical University, Taichung, Taiwan; 8 School of Medicine, National Yang-Ming University, Taipei, Taiwan; 9 National Defense Medical Center, Taipei, Taiwan; 10 Department of Nursing, College of Health, National Taichung University of Science and Technology, No. 1650 Taiwan Boulevard Sect. 4, Taichung 40705, Taiwan

**Keywords:** Iliopsoas abscess, Percutaneous drainage, Psoas abscess, Pyomyositis, Surgery

## Abstract

**Background:**

Percutaneous drainage (PCD) and surgical intervention are two primary treatment options for iliopsoas abscess (IPA). However, there is currently no consensus on when to use PCD or surgical intervention, especially in patients with gas-forming IPA. This study compared the characteristics of patients with gas-forming and non-gas forming IPA and their mortality rates under different treatment modalities. An algorithm for selecting appropriate treatment for IPA patients is proposed based on our findings.

**Methods:**

Eighty-eight IPA patients between July 2007 and February 2013 were enrolled in this retrospective study. Patients < 18 years of age or with an incomplete course of treatment were excluded. Demographic information, clinical characteristics, and outcomes of different treatment approaches were compared between gas-forming IPA and non-gas forming IPA patients.

**Results:**

Among the 88 enrolled patients, 27 (31%) had gas-forming IPA and 61 (69%) had non-gas forming IPA. The overall intra-hospital mortality rate was 25%. The gas-forming IPA group had a higher intra-hospital mortality rate (12/27, 44.0%) than the non-gas forming IPA group (10/61, 16.4%) (*P* < 0.001). Only 2 of the 13 patients in the gas-forming IPA group initially accepting PCD had a good outcome (success rate = 15.4%). Three of the 11 IPA patients with failed initial PCD expired, and 8 of the 11 patients with failed initial PCD accepted salvage operation, of whom 5 survived. Seven of the 8 gas-forming IPA patients accepting primary surgical intervention survived (success rate = 87.5%). Only 1 of the 6 gas-forming IPA patients who accepted antibiotics alone, without PCD or surgical intervention, survived (success rate = 16.7%). In the non-gas forming IPA group, 23 of 61 patients initially accepted PCD, which was successful in 17 patients (73.9%). The success rate of PCD was much higher in the non-gas forming group than in the gas-forming group (*P* <0.01).

**Conclusions:**

Based on the high failure rate of PCD and the high success rate of surgical intervention in our samples, we recommend early surgical intervention with appropriate antibiotic treatment for the patients with gas-forming IPA. Either PCD or primary surgical intervention is a suitable treatment for patients with non-gas forming IPA.

## Background

Iliopsoas abscess (IPA), a suppurative collection within the compartment of the psoas and iliacus muscles, was first reported by Mynter et al. in 1881 [1]. It is an infrequent but potentially life-threatening infectious disease. Prior to introduction of effective anti-tuberculosis drugs, IPA was once a common complication of tuberculous spinal infection. With the common application of anti-tuberculosis drugs, non-tuberculous pyogenic IPA has become the predominant form. Clinically, IPA is classified according to its origin [2]. Primary IPA is thought to be secondary to an unrecognized staphylococcal bacteremia, while secondary IPA is caused by underlying conditions such as gastrointestinal or genitourinary tract diseases, spread of infection from postoperative aortic aneurysm, spondylodiskitis, osteomyelitis, septic arthritis, and infection subsequent to renal surgery [1,3]. Primary IPA is often seen in younger patients and in developing or tropical countries, while secondary IPA occurs more frequently in developed countries with mixed enteric flora [2]. In recent decades, the incidence of primary IPA has gradually increased as numbers of intravenous drug-abusers and those afflicted with human immunodeficiency virus (HIV) infection increase [1,4].

The *psoas-muscle sign*, the triad of fever, flank pain, and limitation of hip movement, is noted in only 30% patients [5]. Because of the rapid progress in advanced imaging techniques, such as gallium-67 scanning, computed tomography (CT), and magnetic resonance image (MRI) in recent years, early diagnosis of IPA has become easier despite initial clinical presentation that is frequently ambiguous [6-8]. Although origin-based IPA classification has been adopted in clinical practice, it does not help clinicians make appropriate treatment decisions. Currently, whether percutaneous drainage (PCD) or surgical intervention should be used in IPA patients remains controversial because few studies with large sample sizes have been performed. At present, PCD is considered preferable to surgical intervention for the treatment of IPA [9-11].

In this study, we compared demographic information, laboratory data, microbiological distribution, origin of infection, treatment modality (i.e., antibiotics alone, PCD, and surgical intervention), and clinical outcomes between patients with gas-forming IPA and non-gas forming IPA. From these results, we established an evidence-based algorithm for selecting an appropriate treatment modality for IPA.

## Methods

This retrospective study was approved by the institutional review board of Taichung Veterans General Hospital (No. CE13129). The data were retrieved from the electronic clinical database of Taichung Veterans General Hospital, a 1,520-bed referral medical center in central Taiwan. The diagnosis of IPA was confirmed by (1) documentation of abscess within the iliopsoas muscle by either PCD or surgery; or (2) classic CT or MRI finding of IPA with compatible clinical presentation, laboratory data, and blood cultures. Typical CT or MRI images in IPA patients included the following features: (1) enlargement of affected muscle, (2) rim enhancement of the abscess wall with lower central attenuation, and (3) gas presence either as an air–fluid interface or mottled air bubbles. All CT and MRI images were reviewed by the primary study investigators and one radiologist.

The primary outcome of this study was the intra-hospital mortality of treated IPA patients. We defined success of treatment as improvement in clinical condition, decrement of IPA size in follow-up images, and discharge alive after treatment with PCD, surgery, or antibiotics alone. Failure of treatment was defined as mortality during hospitalization or deterioration of a patient’s clinical condition with accompanying non-decrement of IPA size on follow-up images necessitating another advanced treatment modality (i.e., antibiotics alone plus PCD, antibiotics alone plus surgery, or PCD followed by surgery). All the patients accepted a minimum follow-up period of 2 months. Cases were excluded if the patient was < 18 years old or had an incomplete treatment course.

Eighty-eight IPA patients admitted to the hospital between July 2007 and February 2013 were included in this study. Demographic data, laboratory studies, etiological pathogens, infection origins, management approaches, clinical courses, and patient’s outcomes were coded for further statistical analysis. Continuous variables were reported as mean ± SD, whereas categorical variables were described as the number and percentage of subjects. Patients were divided into gas-forming IPA and non-gas forming IPA groups. Further comparisons were performed using Mann–Whitney U test for continuous variables and chi-square test or Fisher’s exact test for categorical variables. A *P* value of less than 0.05 was accepted as statistically significant. Statistical analysis was performed using the Statistical Package for the Social Sciences Version 15.1 (SPSS Inc., Chicago, IL, USA).

## Results

### Clinical features and biochemistry

Of the 88 IPA patients enrolled (58 females, 30 males; mean age 63.0 ± 15.6 years [range, 29–93 years]), 29 patients (33.0%) had bilateral IPA and 74 (84.1%) had multiloculated IPA determined on CT. Mean maximal transverse IPA cavity diameter on CT was 3.98 ± 2.31 cm. Fever or hypothermia was noted in 74 patients (84.0%) upon initial presentation to the hospital. IPA was of primary origin in 21 patients (24%) and of secondary origin in 67 (76%). Severe sepsis was noted in 53 patients (60.2%) upon initial presentation. The average length of hospital stay was 36 ± 39 days (range, 2–328 days; median, 28 days) and the intra-hospital mortality rate was 25%.

### Etiological pathogens

The microbiological documentation rate of all IPA patients via either pus or correlated blood cultures was 83.0% (73/88), including 59.3% (51/86) with positive blood culture, and 75.7% (53/70) with positive pus culture. *Staphylococcus* spp*.* were the most common pathogens in this study, found in blood cultures in 27 patients and pus cultures in 26; followed by *Streptococcus* spp.; *Klebsiella pneumoniae*; and *Escherichia coli*. Methicillin-resistant *Staphylococcus aureus* (MRSA) was found in blood cultures in 10 patients and pus cultures in 9 patients. Anaerobic blood cultures were positive in 7 patients and anaerobic pus cultures were positive in 13 patients. Only one pus culture was positive for tuberculosis.

### Origins of secondary IPA

Among the 61 secondary IPA patients, the most common etiologies were skeletal (37 patients, 60.7%), followed by cardiovascular (11 patients, 18.0%) and urinary tract (10 patients, 16.4%) (Table 1). Cardiovascular origins of IPA included 3 cases (4.9%) of infective endocarditis with distal septic infection, 4 cases (6.6%) of abdominal aortic aneurysm with subsequent infection after endovascular stent implantation, and 2 cases (3.3%) of infected aortic aneurysm.

**Table 1:** Classification of IPA origin between gas-forming IPA and non-gas forming IPA patients

	**Gas-forming IPA (n = 27)**	**Non-gas forming IPA (n = 61 )**
Origin (Primary:Secondary)	3:24	18:43
**Skeletal**	11 (40.7%)	26 (42.6%)
Vertebral osteomyelitis/spondylodiskitis	10	17
Ilio-sacral joint septic arthritis	0	7
Hip septic arthritis	1	2
**Intra-abdominal**	1 (3.7%)	4 (6.6%)
Necrotizing pancreatitis	0	2
Appendicitis	0	1
Colon cancer with abscess	1	0
Post GI procedure	0	1^a^
**Cardiovascular**	6 (22.2%)	5 (8.2%)
Abdominal aortic aneurysm post-stent implantation	2	2
Infected aortic aneurysm	2	0
Infective endocarditis	0	3
Catheter related	2	0
**Urinary tract**	5 (18.5%)	5 (8.2%)
Acute pyelonephritis	2^b^	2^c^
Post GU procedure	3^d^	2^e^
Prostate abscess	0	1
**Post gynecologic procedure**	0	1^f^ (1.6%)
**Empyema with downward extension**	0	2 (3.3%)
**Limb necrotizing fasciitis**	1 (3.7%)	0

^a^Post appendectomy and colocutaneous fistula repair.

^b^Including 2 EPN.

^c^ Including 1 EPN.

^d^Including 1 post nephrectomy, 1 post Bench surgery, and 1 renal stone post ESWL.

^e^Including 2 post nephrectomy.

^f^Endometrioid cancer post operation.

EPN, emphysematous pyelonephritis; ESWL, extracorporeal shock wave lithotripsy; GI, gastrointestinal; GU, genitourinary.

### Imaging studies

CT confirmed the diagnosis of IPA in all patients. MRI was performed in 20 patients with poor treatment response or high suspicion for infection of adjacent structures. Accordingly, osteomyelitis/spondylodiskitis and epidural abscess were demonstrated in 18 (20.5%) and 11 (12.5%) patients, respectively. The 11 patients with epidural abscess underwent subsequent surgical intervention and 3 expired.

### Causes of mortality

The ultimate causes of mortality of our patients were examined by two research physicians via review of medical records. In controversial cases, the primary care physicians were consulted and consensus was reached after thorough discussion. There were 22 mortality cases in our study group, and 16 cases (72.7%) expired due to refractory sepsis. Six cases (27.3%) expired because of comorbidities, including 2 from massive upper gastrointestinal bleeding and 2 from hemorrhagic shock caused by aortic aneurysm rupture. The causes of mortality of the remaining 2 cases were unexpected cardiac arrest and end-stage cancer.

### Initial treatment options

Once the patients with suspicious infection presented to the hospital, empirical antibiotics were prescribed within 2 hours. Third-generation cephalosporin plus metronidazole (15/88, 17.0%) or carbapenem (13/88, 14.8%) were most often prescribed initially to our study cases. Antibiotics were adjusted after demonstration of IPA via advanced image studies or available blood/pus cultures. PCD and surgery were the standard treatment modalities for IPA if drainage was indicated. Patients who did not require (because the condition was mild) or were not candidates for (because the condition was too severe for invasive treatment) PCD or surgery received therapy with antibiotics alone.

A comparison of characteristics of IPA patients between different initial treatment modalities (antibiotics alone, PCD, or surgery) is shown in Table 2. Patients who underwent antibiotic therapy alone were older (*P* = 0.005) and had smaller mean diameter of the IPA cavity (*P* < 0.0001) and lower platelet count (*P* = 0.016) than patients receiving other forms of treatment. Impaired renal function was more frequent in this group (*P* < 0.05). Shorter length of hospital stay (*P* = 0.001) and higher mortality rate (*P* = 0.002) were also observed. Other variables were not different between the three groups. Initial treatment with antibiotics alone was chosen in our study group when the IPA was too small to be drained by PCD (n = 9) or when patients were too weak to undergo an invasive procedure (n = 15).

**Table 2:** Comparison of patient characteristics between initial treatment with antibiotics alone, PCD, or surgery

	**Antibiotics (n = 24)**	**PCD (n = 36)**	**Surgery (n = 28)**	***P *** **value**
	**Mean±SD**	**Mean±SD**	**Mean±SD**	
Age (years)	72.0±13.8	59.6±15.6	60.0±14.6	0.005*
Sex (F:M)	15:9	23:13	20:8	0.752
Gas-forming IPA	18 (75.0%)	23 (63.9%)	20 (71.4%)	0.631
Bilateral	10 (41.7%)	10 (27.8%)	9 (32.1%)	0.530
Multiloculated IPA collection pattern	17 (70.8%)	33 (91.7%)	24(85.7%)	0.093
Mean maximal transverse IPA cavity diameter on CT (cm)	2.2 ±1.5	5.2 ±2.1	4.0±2.2	<0.0001*
DM	10 (41.7%)	13 (36.1%)	11 (39.3%)	0.907
Hypertension	8 (33.3%)	12 (33.3%)	13 (46.4%)	0.497
CKD	9 (37.5%)	7 (19.4%)	3 (10.7%)	0.060
Malignancy	4 (16.7%)	9 (25.0%)	4 (14.3%)	0.520
Liver cirrhosis	4 (16.7%)	6 (16.7%)	2 (7.1%)	0.479
Fever or hypothermia	20 (83.3%)	31 (86.1%)	23 (82.1%)	0.905
Severe sepsis	18 (75.0%)	29 (80.6%)	21 (75.0%)	0.830
WBC (/μL)	18900±9428	17672 ±9018	15354±7426	0.344
Bandemia (No. of patients)	12 (50.0%)	17 (47.2%)	9 (32.1%)	0.353
Hb (g/dL)	10.8±2.4	10.7 ±2.3	10.7±2.7	0.996
Platelet (×10^3^/μL)	185.5±140.9	309.2 ±179.0	255.6±144.8	0.016*
BUN (mg/dL)	65.6±67.1	36.8 ±37.1	32.4±28.0	0.041*
Creatinine (mg/dL)	3.5±4.0	2.2 ±2.0	1.43±0.9	0.037*
ALT (U/L) (n = 24 vs 35 vs 28)	106.3±250.6	51.0 ±77.7	80.1±244.9	0.768
Na (mEq/L)	132.3±8.5	133.4 ±5.6	135.0±5.5	0.258
K (mEq/L)	4.4±1.0	4.0 ±0.5	4.1±0.7	0.223
CRP (mg/L)	16.0±10.5	21.9 ±10.1	19.3±12.2	0.083
CK (U/L) (n = 22 vs 29 vs 25)	539.3±1711.5	175.6 ±302.3	183.1±535.1	0.309
Initial blood glucose (mg/dL) (n = 24 vs 34 vs 28)	180.0±116.0	208.0 ±151.3	155.3±88.6	0.369
Primary:Secondary origin	8:16	8:28	5:23	0.408
Mortality	12 (50.0%)	8 (22.2%)	2 (7.1%)	0.002*
Length of hospital stay (days)	19.4±15.2	44.9 ±55.3	38.8±19.5	0.001*

*P < 0.05.

ALT, alanine aminotransferase; BUN, blood urea nitrogen; CK, creatine kinase; CKD, chronic kidney disease; CRP, C-reactive protein; CT, computed tomography; DM, diabetes mellitus; Hb, hemoglobin; IPA, iliopsoas abscess; K, potassium; Na, sodium; PCD, percutaneous drainage; SD, standard deviation; WBC, white blood cells.

### Factors contributing to mortality

Possible contributions of age, sex, clinical and laboratory variables to mortality were analyzed using univariate analysis (Table 3). Results revealed that age, gas-forming IPA, white blood cell (WBC) count, platelet count, blood urea nitrogen (BUN), creatinine, potassium, and secondary IPA origin were significantly associated with mortality. Further Cox regression analysis of these variables as well as bilateral IPA revealed that none was associated with mortality rate except initial treatment. The hazard ratio (HR) for treatment using antibiotics alone was 5.34 (95% confidence interval [CI], 1.27–22.56), whereas surgery had no effect on mortality rate (HR = 0.28, 95% CI 0.04–2.02, *P* = 0.207).

**Table 3:** Univariate and multivariate analyses of effects of age, sex and clinical variables on mortality rate

	**Univariate analysis**			**Multivariate analysis**^**a**^		
	**HR**	**95% CI**	***P *** **value**	**HR**	**95% CI**	***P *** **value**
Age (years)	1.05	(1.010-1.083)	0.013*	1.03	(0.986-1.071)	0.204
Sex-female	1.06	(0.421-2.655)	0.905	1.21	(0.369-3.997)	0.749
Gas-forming IPA	2.57	(1.060-6.250)	0.037*	1.93	(0.425-8.804)	0.393
Bilateral IPA	2.28	(0.943-5.524)	0.067	1.76	(0.563-5.481)	0.332
Multiloculated IPA collection pattern	0.85	(0.248-2.938)	0.801			
Mean maximal transverse IPA cavity diameter on CT (cm)	0.93	(0.747-1.162)	0.533			
DM	1.21	(0.490-2.996)	0.679			
Hypertension	0.41	(0.145-1.134)	0.085			
CKD	1.22	(0.459-3.265)	0.687			
Malignancy	1.34	(0.442-4.057)	0.605			
Liver cirrhosis	1.19	(0.346-4.080)	0.785			
Fever or hypothermia	1.53	(0.351-6.647)	0.572			
Severe sepsis	29.17	(0.215-3952)	0.178			
WBC (/μL)	1.00	(1.000-1.000)	0.004*	1.00	(1.000-1.000)	0.109
Bandemia	1.76	(0.722-4.315)	0.213			
Hb (g/dL)	0.89	(0.737-1.084)	0.255			
Platelet (×10^3^/μL)	1.00	(0.992-0.999)	0.013*	1.00	(0.992-1.001)	0.109
BUN (mg/dL)	1.01	(1.005-1.017)	0.001*	1.00	(0.983-1.010)	0.623
Creatinine (mg/dL)	1.12	(1.009-1.233)	0.033*	0.93	(0.738-1.178)	0.555
ALT (U/L)	1.00	(0.998-1.002)	0.702			
Na (mEq/L)	1.01	(0.946-1.083)	0.721			
K (mEq/L)	2.54	(1.442-4.485)	0.001*	1.36	(0.674-2.761)	0.389
CRP (mg/L)	0.98	(0.935-1.021)	0.303			
CK (U/L)	1.00	(0.999-1.001)	0.972			
Initial blood glucose (mg/dL)	1.00	(0.996-1.003)	0.750			
Secondary IPA origin	0.37	(0.149-0.931)	0.035*	0.35	(0.081-1.490)	0.154
Initial treatment						
Antibiotics alone	6.49	(2.132-19.753)	0.001*	5.34	(1.266-22.558)	0.023*
PCD	Reference			Reference		
Surgery	0.51	(0.098-2.627)	0.418	0.28	(0.039-2.017)	0.207

^a^adjusted for age, gender, gas-forming IPA, bilateral IPA, WBC, Platelet, BUN, creatinine, K, secondary IPA origin, and initial treatment.

*P < 0.05.

ALT, alanine aminotransferase; BUN, blood urea nitrogen; CK, creatine kinase; CKD, chronic kidney disease; CRP, C-reactive protein; CT, computed tomography; DM, diabetes mellitus; Hb, hemoglobin; HR, hazard ratio; IPA, iliopsoas abscess; K, potassium; Na, sodium; PCD, percutaneous drainage; SD, standard deviation; WBC, white blood cells.

### Comparison between patients with gas-forming and non-gas forming IPA

Results of comparisons between gas-forming IPA and non-gas forming IPA patients are summarized in Table 4. Among the 88 patients enrolled, 27 (30.7%) had gas-forming IPA and 61 (69.3%) had non-gas forming IPA. No sex predominance between groups was identified. Patients in the gas-forming IPA group showed a higher incidence of bilateral involvement (14/27, 51.9%) than the non-gas forming IPA group (15/61, 24.6%) (*P* < 0.05). Neither the incidence of multiloculated IPA collection pattern (92.5% versus 80.3%) nor the mean maximal transverse IPA cavity diameter (4.21 cm versus 3.87 cm) was significantly different between the 2 groups. The gas-forming IPA group had a higher intra-hospital mortality rate (12/27, 44.0%) than the non-gas forming IPA group (10/61, 16.4%) (*P* < 0.001). No difference in underlying disease, primary versus secondary origin, biochemistry profile, or average length of hospital stay was found between the 2 groups.

**Table 4:** Comparison between gas-forming IPA and non-gas forming IPA patients

	**Gas-forming IPA**	**Non-gas forming IPA**	***P *** **value**
	**(n = 27)**	**(n = 61 )**	
	**Mean±SD**	**Mean±SD**	
Age (years)	67.8±13.9	61.1±16.0	0.053
Sex (F:M)	14:13	44:17	0.064
Bilateral	14(51.9%)	15(24.6%)	0.012*
Multiloculated IPA collection pattern^f^	25(92.6%)	49(80.3%)	0.211
Mean maximal transverse IPA cavity diameter on CT (cm)	4.21±1.79	3.87±2.51	0.166
DM	13(48.2%)	21(34.4%)	0.223
Hypertension	8(29.6%)	25(41.7%)	0.284
Uremia^f^	3(11.1%)	8(13.1%)	1.000
CKD	5(18.5%)	14(23.0%)	0.641
Malignancy	4(14.8%)	13(21.7%)	0.456
Liver cirrhosis^f^	1(3.7%)	11(18.0%)	0.096
Fever or hypothermia^f^	26(96.3%)	48(78.7%)	0.056
	(Including 2 hypothermia)	(Including 4 hypothermia)	
Severe sepsis	27(100.0%)	26(67.2%)	0.002*
WBC (/μL)	20363±11077	15900±7059	0.132
Bandemia (No. of patients)	15(55.6%)	23(37.7%)	0.119
Hb (g/dL)	10±2.4	11±2.4	0.056
Platelet (×10^3^/μL)	244.7±205.2	264.5±145.0	0.234
BUN (mg/dL)	55.4±55.0	37.9±41.9	0.053
Creatinine (mg/dL)	2.5±2.1	2.2±2.8	0.158
ALT (U/L) (n = 27 vs 60)	68.5±110.4	78.8±225.3	0.287
Na (mEq/L)	132.3±6.5	134.2±6.5	0.132
K (mEq/L)	4.3±0.8	4.1±0.7	0.178
CRP (mg/L)	217.8±92.8	184.8±116.9	0.168
CK (U/L) (n = 22 vs 54)	207.4±360.3	314.3±1150.0	0.439
Initial blood glucose (mg/dL) (n = 27 vs 59)	211.4±139.9	170.0±115.9	0.098
Primary:Secondary origin	3:24	18:43	0.062
Mortality	12(44.4%)	10(16.4%)	0.005*
Length of hospital stay (days)	37.6±32.8	35.3±41.6	0.906

^f^Fisher’s Exact Test. *P < 0.05.

ALT, alanine aminotransferase; BUN, blood urea nitrogen; CK, creatine kinase; CKD, chronic kidney disease; CRP, C-reactive protein; CT, computed tomography; DM, diabetes mellitus; Hb, hemoglobin; IPA, iliopsoas abscess; K, potassium; Na, sodium; SD, standard deviation; WBC, white blood cells.

The overall microbiological documentation rate was 85.2% (23/27) in patients with gas-forming IPA and 82.0% (50/61) in patients with non-gas-forming IPA. The distribution of pathogens in blood and pus cultures is summarized in Table 5. The predominant etiological micro-organisms in the non-gas forming IPA group were gram-positive cocci (GPC); mainly *Staphylococcus* spp. The incidences of GPC and gram-negative bacilli (GNB) were equal in the gas-forming IPA group. The incidence of GNB in blood or pus cultures was higher in the gas-forming IPA group than in the non-gas forming IPA group (51.9% versus 27.9%) (*P* < 0.05). The same trend was found for the anaerobes (37.0% versus 14.8%) (*P* < 0.05).

**Table 5:** Distribution of pathogens between gas-forming IPA and non-gas forming IPA patients

	**Gas-forming n = 27**		**Non-gas forming n = 61**	
	**Blood**	**Pus**	**Blood**	**Pus**
GPC	10 (37.0%)	12 (44.4%)	30 (49.2%)	28 (45.9%)
*Staphylococcus* spp.	6	6	21	20
MRSA	3	2	7	7
OSSA	3	3	11	11
Coagulase-negative *Staphylococcus*	0	1	3	2
*Streptococcus* spp.	4	4	8	6
*Enterococcus*	0	2	1	2
GNB	9 (33.3%)	14 (51.9%)	6 (9.8%)	13 (21.3%)
*Escherichia coli*	1	5	1	4
*Klebsiella pneumoniae*	3	4	0	6
Others				
Anaerobics	3 (11.1%)	9 (33.3%)	4 (6.6%)	6 (9.8%)
Fungus	0	1 (3.7%)	2 (3.3%)	3 (4.9%)
TB	0	0	0	1 (1.6%)
Mixed flora	3 (11.1%)	7 (25.9%)	8 (13.1%)	11 (18.0%)
MDR isolates	2 (7.4%)	3 (11.1%)	0	3 (4.9%)

GNB, Gram-negative bacilli; GPC, Gram-positive cocci; IPA, iliopsoas abscess; MDR, multiple-drug resistant; MRSA, methicillin-resistant *Staphylococcus aureus*; OSSA, oxacillin-sensitive *Staphylococcus aureus.*

**Figure 1:**
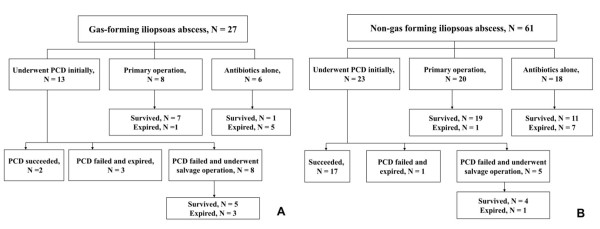
**Management of patients with gas-forming (A) and non-gas forming (B) IPA.** IPA, iliopsoas abscess.

**Figure 2:**
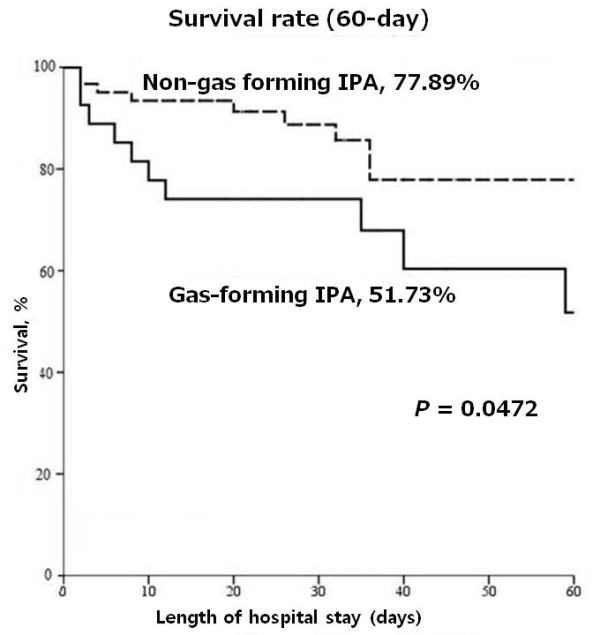
**Kaplan-Meier analysis of cumulative survival rate during a 60-day hospital stay between patients with gas-forming and non-gas forming IPA.** IPA, iliopsoas abscess.

All patients in this study were initially treated with empirical antibiotics. Only 2 among the 13 gas-forming IPA patients initially undergoing PCD had a successful outcome (success rate = 15.4%); 3 of the 11 IPA patients with failed initial PCD expired; and 8 of the 11 patients with failed initial PCD underwent salvage operation, of whom 5 survived. Seven of the 8 gas-forming IPA patients who underwent primary surgical intervention survived (success rate = 87.5%). Six gas-forming IPA patients were treated with antibiotics alone without PCD or surgical intervention, only 1 of whom survived (success rate = 16.7%). In the non-gas forming IPA group, 23 of 61 patients initially underwent PCD; success was achieved in 17 patients (success rate = 73.9%). The success rate of PCD was higher in the non-gas forming than in the gas-forming IPA group (*P <*0.01) (Table 6). Nineteen of 20 non-gas forming IPA patients undergoing primary operation survived. The survival rates of patients who received antibiotics alone in the gas-forming IPA and non-gas forming IPA groups were 16.7% (1/6) and 61.1% (11/18), respectively. The treatment approaches in the gas-forming IPA and non-gas forming IPA groups are delineated in Figure 1. We also conducted a Kaplan-Meier analysis to compare the survival rate within 60 days between the 2 groups. Log rank test showed a higher survival rate in the non-gas forming IPA group (77.9%) than in the gas-forming group (51.7%) (*P* < 0.05) (Figure 2).

**Table 6:** Success rate of different management pathways between gas-forming IPA and non-gas forming IPA patients

	**Gas-forming IPA**	**Non-gas forming IPA**	***P *** **value**
	**(n = 27)**	**(n = 61 )**	
Total number of initial treatments with PCD	13 (48.2%)	23 (37.7%)	0.358
PCD success rate	2/13 (15.4%)	17/23 (73.9%)	0.0007**
Total number undergoing surgical debridement	16 (59.3%)	25 (41.0%)	0.113
Primary operation success rate^f^	7/8 (87.5%)	19/20 (95.0%)	0.497
Salvage operation success rate (in patients with failed PCD)^f^	5/8 (62.5%)	4/5 (80.0%)	1.000
Antibiotics alone success rate^f^	1/6 (16.7%)	11/18 (61.1%)	0.155

^f^Fisher’s Exact Test. **P < 0.001.

## Discussion

Iliopsoas muscles are susceptible to infections from distant sites and contiguous structures because of their rich blood supply and overlying retroperitoneal lymphatic systems. IPA is often overlooked because of its insidious onset, and subsequent severe sepsis may be life-threatening. Although the exact incidence of IPA is unknown, more and more cases are being identified because of widespread application of CT in suspected IPA patients. Despite recent advances in diagnostic modalities, the mortality rate of IPA has not improved much (6.7% in a study by Ricci in 1986 and 5% in a study by Navarro in 2009) [2,12]. In our study, the mortality rate in IPA patients (22/88, 25%) was higher than that reported in a meta-analysis conducted by Lai et al. (8%, 55/682, between 1986 and 2011) [9]. However, because those patients were from various medical settings in different countries, the results of that meta-analysis cannot be used as the gold standard for clinical comparison, and further clarification is needed. Among the 88 patients in our study group, 24 (27.3%) had already been hospitalized in other hospitals and were referred to us because of poor treatment response. The mortality rate among these patients was high (7/24, 29.2%). The remaining patients (64/88, 72.7%) were admitted via our emergency department (ED) with initial severe illness. In addition, severe sepsis was frequently observed in our total study group. The general poor health of the patients was probably the major cause of high mortality in the study group. In addition, at our hospital, more conservative treatment policies were adopted because of patients’ older age, which itself might have contributed to a higher mortality rate. Other possible explanations of the high mortality rate include large number of gas-forming IPA cases and predominance of secondary IPA.

The presentations of IPA in our study varied, including fever, flank pain or back pain, abdominal pain, thigh lumps, altered consciousness, and even shock. The initial nonspecific symptoms; e.g., nausea, general weakness, intermittent mild fever, or low back soreness, might easily be ignored by patients. We attempted to evaluate how long the patients had been ill via a complete medical record review. Pre-presentation symptoms could not be evaluated definitively in 24 of 88 patients (27.3%) who had stayed in other hospitals for periods of time. A higher mortality rate (7/24, 29.2%) was noted in this group. Additionally, the morphology of IPA pattern, according to the images in our hospital, might have been influenced by prior therapy. Sixty-four patients (72.7%) in our study were directly referred from another ED because of severe illness or initially sought medical assistance in our hospital. The majority of patients in this group (55/64, 85.9%) had a long illness prior to presentation; 12.55 days on average. Among these 55 patients, 17 (30.9%) had bilateral IPA and 49 (89.1%) had multiloculated IPA. The remaining 9 patients (9/64, 14.1%) in this group could not be evaluated because medical records were unclear or because patients had deterioration of consciousness upon initial presentation and ultimately expired. In brief, the late presentation of most of our patients may have contributed to the high total mortality.

IPA is clinically classified into primary and secondary origins according to its initial infection site. In 1986, Ricci et al. described 286 cases of primary IPA, mainly young patients from developing countries [2]. There were 90 cases of secondary IPA, which were almost exclusively from developed countries, and the most common etiologies were Crohn’s disease, followed by appendicitis and colon inflammation or cancer. Since then, more and more patients have been found to have secondary rather than primary IPA. In 2009, Navarro et al. reported 124 cases in a multi-center study, 78.2% with secondary IPA, and a single-center study by Carolyn et al. in 2001 demonstrated 80% of 61 cases having secondary IPA. The main sources of secondary IPA in the study by Navarro et al. were skeletal (50.5%), followed by gastrointestinal tract (24%) and genitourinary tract (17.5%). Our study revealed a similar trend with predominance of secondary IPA (76.1%) in which the most common infection origins were skeletal, followed by cardiovascular system and urinary tract. The higher prevalence of secondary IPA reported recently and in our study may be attributable to the widespread application of CT and MRI [13]. These imaging modalities can clearly delineate adjacent structures, especially the vertebrae and epidural space, and it is therefore likely that the infectious focus will be clarified. Furthermore, in our report, 11 patients of secondary IPA originated from cardiovascular system, including abdominal aortic aneurysm (AAA) post-stent implantation with subsequent infection, infected aortic aneurysm, and infective endocarditis, all of which have rarely been mentioned in the literature [14,15].

Gas-formation is an important predictor of clinical outcome for patients with liver abscess and acute pyelonephritis [16,17]. Furthermore, in patients with acute pyelonephritis, the treatment policy depends mainly on whether the gas-forming (emphysematous) change is detected within the urinary tract. The major components of the gas in emphysematous pyelonephritis are nitrogen, hydrogen, carbon dioxide, and oxygen, and mixed acid fermentation has been proposed as the major mechanism of gas production because of the hydrogen content [17,18]. Other authors have indicated that rapid tissue catabolism complicated by impaired transport of end products around the infection site produces the gas [19]. Based on the clinical consensus that in acute pyelonephritis gas formation is the major criterion for further treatment decisions, we propose a new algorithm for determining treatment modality in IPA patients according to the presence of gas. In our study, GNB and anaerobic infections were more often discovered in patients with gas-forming IPA than in those with non-gas forming IPA. This phenomenon may explain the mechanism of gas formation and the development of a fulminant course of IPA.

**Figure 3:**
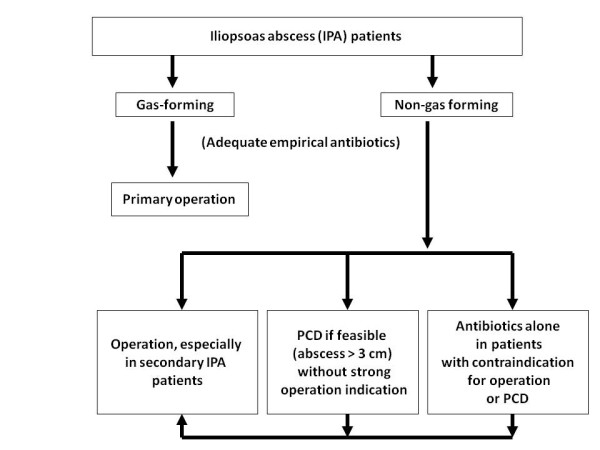
**Suggested clinical management algorithm for IPA patients.** IPA, iliopsoas abscess.

Abscess drainage plus antibiotic treatment is essential for appropriate management of IPA patients. Options for drainage are surgery or PCD. Generally, IPA patients with concurrent intra-abdominal or retroperitoneal abnormalities such as ruptured appendicitis or ruptured infected aortic aneurysm require surgery to clean out or repair the infected foci. Surgical intervention may provide more effective drainage than PCD, especially in patients with multiloculated IPA [4]. IPA patients were often treated with surgical debridement in the 1980s, when imaging techniques were still uncommon [2]. Afaq et al. also reported no mortality in 72 cases in Nepal in which all of the patients received surgical intervention as the first choice of treatment [20]. Recent studies have suggested that IPA can be successfully treated with antibiotics plus PCD [11,21], but not all of the cases in our study could be cured by PCD, especially when the IPA was gas-forming. If there are strong indications for primary operation, such as ruptured infected aortic aneurysm, ruptured appendicitis, or epidural abscess with spinal cord compression, surgical intervention should not be delayed. Furthermore, in our study, surgical intervention was preferred when gas-forming IPA was observed because of the higher failure rate of PCD. However, PCD plus appropriate antibiotics is adequate for treating patients with non-gas forming and solitary IPA. In addition, PCD remains an option for IPA patients who are not suitable for operation under general anesthesia. The suggested treatment algorithm based on the results of our study is delineated in Figure 3.

The major strength of this study is the use of a large number of IPA patients from a single center. Although previous multi-center study used larger sample sizes, many relevant variables could not be evaluated because of differences in record format across medical centers. However, there are several limitations to this study. First, there is no international consensus for IPA treatment; treatment policy depends on the treating physicians, whose training and clinical experience may play roles. Second, no matter which modality of treatment is chosen, the origin of infection may affect the clinical outcome, and this could not be evaluated in this study. Third, recurrence was not evaluated in this study. Thus, it is unknown whether repeated PCD or surgical intervention was performed. Fourth, the complexity of the disease varied in our sample.

## Conclusions

Our findings suggest that classification of IPA patients into gas-forming IPA or non-gas forming IPA groups may help physicians make early and definitive decisions with regard to treatment choices. Our study showed that PCD treatment for patients with gas-forming IPA resulted in a high failure rate and increased intra-hospital mortality rate. We recommend early surgical intervention with appropriate antibiotic treatment for gas-forming IPA patients. PCD is adequate for the management of patients with solitary and non-gas forming IPA, especially for primary IPA.

## Competing interests

The authors declare they have no competing interests.

## Authors’ contributions

M-SH, J-AH, and S-YH participated in study design. M-SH, S-CH, Y-YH, C-AT, and Y-TT participated in data acquisition, analysis, and interpretation. Critical revision of the manuscript for important intellectual content: M-SH, E-WL, L-MW, and S-YH. All authors read and approved the final manuscript.

## Pre-publication history

The pre-publication history for this paper can be accessed here:

http://www.biomedcentral.com/1471-2334/13/578/prepub
